# A *Bacillus licheniformis* Glycoside Hydrolase 43 Protein Is Recognized as a MAMP

**DOI:** 10.3390/ijms232214435

**Published:** 2022-11-20

**Authors:** Zhixiang Yuan, Ying Zhao, Zhitong Mo, Hongxia Liu

**Affiliations:** 1 College of Plant Protection, Nanjing Agricultural University, Nanjing 210095, China; 2 Key Laboratory of Integrated Management of Crop Diseases and Pests, Ministry of Education, Nanjing Agricultural University, Nanjing 210095, China

**Keywords:** *Bacillus licheniformis*, protein elicitor, glycoside hydrolase 43 family, induced systemic resistance

## Abstract

Glycoside hydrolases from pathogens have often been reported as inducers of immune responses. However, the roles of glycoside hydrolase from plant-growth-promoting rhizobacteria (PGPR) in the resistance of plants against pathogens is not well studied. In this study, we identified a glycoside hydrolase 43 protein, H1AD43, produced by *Bacillus licheniformis* BL06 that can trigger defense responses, including cell death. Ion-exchange and size-exclusion chromatography were used for separation, and the amino acid sequence was identified by mass spectrometry. The recombinant protein generated by prokaryotic expression was able to elicit a hypersensitive response (HR) in *Nicotiana benthamiana* and trigger early defense responses, including reactive oxygen species (ROS) burst, callose accumulation, and the induction of defense genes. In addition, the protein could induce resistance in *N. benthamiana*, in which it inhibited infection by *Phytophthora capsici* Leonian and tobacco mosaic virus-green fluorescent protein (TMV-GFP) expression. H1AD43 thus represents a microbe-associated molecular pattern (MAMP) of PGPR that induces plant disease resistance and may provide a new method for the biological control of plant disease.

## 1. Introduction

During long-term plant-microbe interactions, plants have evolved a series of defenses against attack by pathogens [1]. There are two layers of the plant immune defense system, including PTI (PAMP-triggered immunity), triggered by cell-surface-localized pattern recognition receptors (PRRs), and ETI (effector-triggered immunity), triggered by sensing effectors in the cytoplasm [2,3]. MAMPs (microbe-associated pattern molecules) are highly conserved among microorganisms and are important for maintaining the basic biological characteristics of microorganisms [4]. When exposed to various stimuli, MAMPs can induce a series of responses in plants, including the production of reactive oxygen species (ROS), deposition of callose, and upregulation of defense-related genes [5]. The microbe-associated molecular patterns discovered in the early stage mainly include flagellin flg22, elongation factor elf18, and glutamyltransferase PEP13 [6,7,8]. Such MAMPs are highly conserved in bacteria and phytophthora, and their mechanism has been studied. In recent years, some protein or nonprotein MAMPs have also been discovered, such as xylanase, cold shock proteins, lipopolysaccharide, and chitin [9,10,11,12].

Glycoside hydrolases (GHs), which are responsible for hydrolyzing glycosidic bonds, are the largest and most diverse protein family in the CAZy database [13]. The plant cell wall is a natural barrier that can prevent the infection of pathogenic bacteria [14]. Its main components are cellulose and pectin [15]. Some pathogenic microorganisms can secrete a large number of cell-wall-degrading enzymes (CWDEs) to degrade the defense barrier of plants during the infection process so that pathogens can invade plant tissues and achieve successful colonization [16]. These CWDEs are generally considered important virulence factors [17], and some CWDEs can also act as MAMPs to activate the immune responses of plants, such as the glycoside hydrolase XEG1 (GH12) produced by *Phytophthora sojae*, which can degrade xylan and β-glucan and acts not only as a virulence factor during *P. sojae* infection but also as a PAMP to induce defense responses in soybean and other solanaceae plants [18]. The xylanase VdEIX3 secreted by *Verticillium dahliae* can cause typical PTI responses by acting as an MAMP, inducing programmed cell death (PCD) in *Nicotiana benthamiana* leaves and causing an ROS burst and upregulation of defense-related genes in *N. benthamiana* leaves [19]. The xylanase BcXyn11A secreted by *Botrytis cinerea* can also act as an elicitor to induce the immune response of plants [20]. In addition to GHs produced by pathogenic microorganisms, GHs produced by biocontrol bacteria also act as elicitors to induce plant defense responses. For example, 1,4-endoxylanase secreted by *Trichoderma viride* can strongly induce the synthesis of pathogenesis-related (PR) proteins in *N. benthamiana* cells, and this elicitor activity is independent of its enzyme activity [21].

However, studies on GHs secreted from *Bacillus* acting as MAMPs to induce plant immunity have rarely been reported. *Bacillus licheniformis* (*Bacillus* sp.) is an important type of plant-growth-promoting rhizobacteria (PGPR) [22]. It has been found that rhizotrophic bacteria can produce protein or nonprotein MAMPs to improve the disease resistance of the host plant. For example, fengycins and surfactins secreted by *Bacillus subtilis* can not only inhibit the growth of pathogenic microorganisms effectively but also act as elicitors to induce plant growth defense responses [23]. PeBA1, a novel protein of *Bacillus amylolyticus* NC6, can induce systemic resistance in *N. benthamiana* [24]. PeBL1, a protein inducer from *Brevibacillus laterosporus* strain A60, activates *N. benthamiana* defense responses [25].

In this study, we found that exoproteins from *B. licheniformis* were able to elicit an HR in *N. benthamiana* leaves, which is a typical disease resistance response [26]. Through the isolation and purification of exoproteins from *B. licheniformis*, we characterized the unreported glycoside hydrolase H1AD43. H1AD43 belongs to the glycoside hydrolase 43 family, which is highly conserved in *Bacillus* and exhibits typical elicitor characteristics. Moreover, H1AD43 could effectively attenuate the infection of plants by pathogenic bacteria. Thus, H1AD43 represents a MAMP of PGPR that improves plant disease resistance.

## 2. Results

### 2.1. Purification, Characterization, and Identification of H1AD43

**Figure 1:**
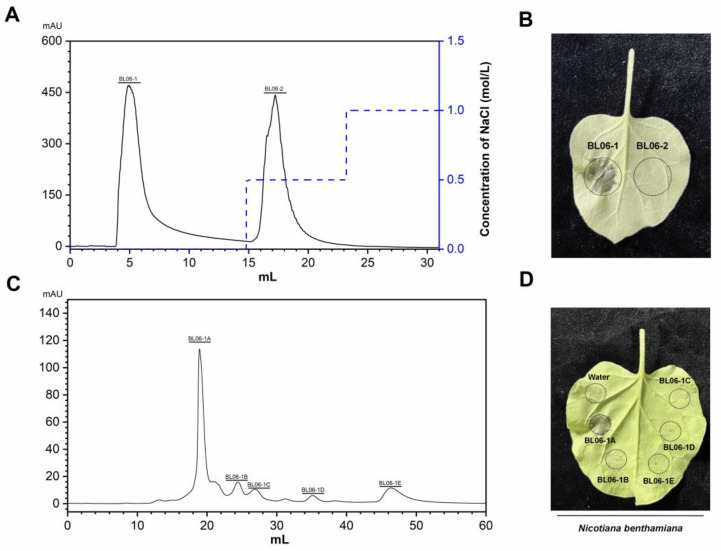
Isolation, purification, and activity verification of the *Bacillus licheniformis* (BL06) exoprotein. (**A**) Elution curve of the BL06 exoprotein by HiTrap Capto Q chromatography; (**B**) Induction of cell death in *Nicotiana benthamiana* leaves by BL06-1 and BL06-2; (**C**) The elution curve of BL06-1 by Superdex75 chromatogram; (**D**) Induction of cell death in *N. benthamiana* leaves by BL06-1A-1E.

**Figure 2:**
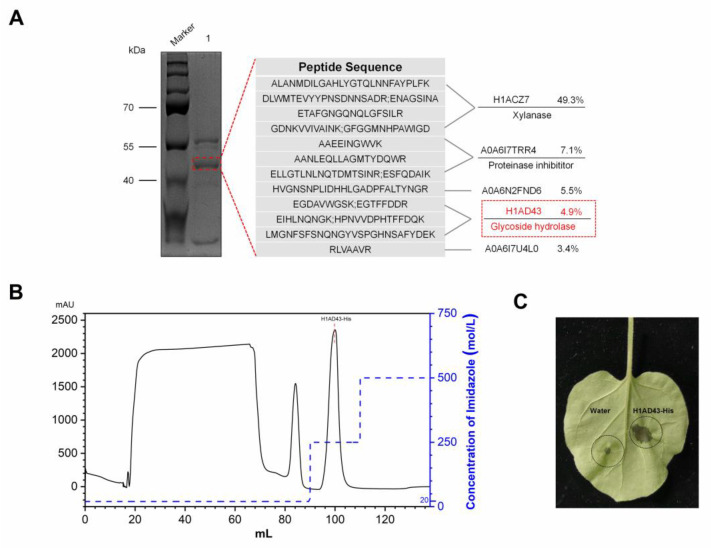
Identification and activity verification of the BL06-1A protein. (**A**) Mass spectrometry analysis of the BL06-1A protein; (**B**) Elution curve of H1AD43 in a His-Trap HP column; (**C**) Induction of cell death in *N. benthamiana* leaves by H1AD43.

When *B. licheniformis* BL06 was inoculated into LB medium for 72 h with shaking, the supernatant of the culture medium triggered the HR in *N. benthamiana* leaves. In contrast, this phenomenon was not observed when the BL06 thallus was infiltrated into *N. benthamiana* (Figure S1). The protein in the culture supernatant was preliminarily separated using ammonium sulfate solution with a saturation of 90%, and the crude BL06-SP protein was obtained and was able to cause an HR in *N. benthamiana* (Figure S2), which suggests that the exoprotein of BL06 plays an important role in the induction of HR. BL06-SP was collected and purified using a DEAE anion column. Two fractions of BL06-SPs were obtained by chromatography: BL06-1, BL06-2, and BL06-1 were able to induce HRs in *N. benthamiana* leaves (Figure 1A,B). BL06-1 was further purified using Superdex75, and finally six purified components (BL06-1A-1E) were obtained. Only BL06-1A could induce an HR in *N. benthamiana* leaves (Figure 1C,D). BL06-1A showed three bands of different molecular weights on SDS-PAGE (Figure 2A), of which we performed mass spectrometry analysis, and according to the alignment of the peptides, the glycoside hydrolase account for a large proportion of BL06-1A (Figure 2A). There have been many reports on the study of glycoside hydrolases in microorganisms as elicitors inducing plant disease resistance. We performed prokaryotic expression of the major glycoside hydrolases obtained by mass spectrometry analysis, evaluated their activities according to the HR, and finally screened the H1AD43 protein, which was able to cause PCD in *N. benthamiana* (Figure 2B,C).

**Figure 3:**
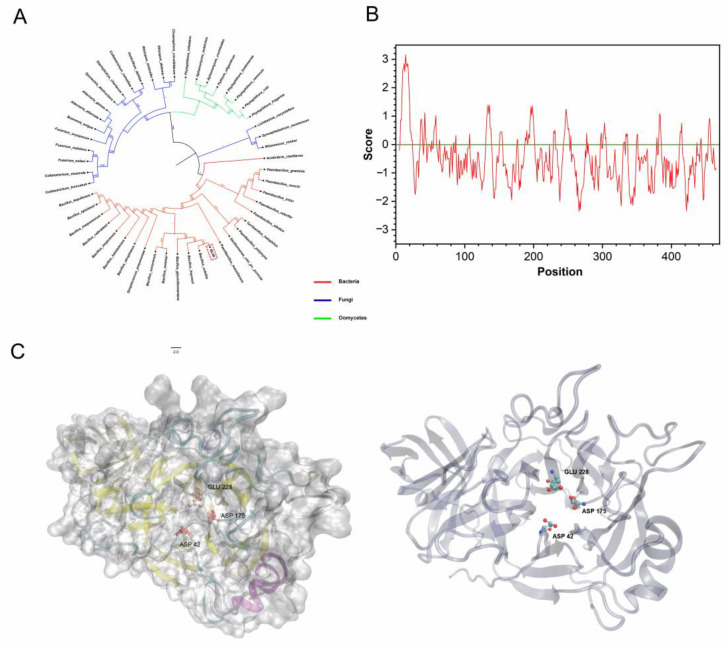
H1AD43 protein property analysis. (**A**) Evolutionary tree analysis of the GH43 family members from different microbial sources; (**B**) H1AD43 hydrophilicity and hydrophobicity analysis; (**C**) H1AD43 structure modeling.

H1AD43 belongs to the GH43 family of glycoside hydrolases and shows xylanohydrolase activity (Figure S3). H1AD43 consists of 469 amino acids with a molecular weight of approximately 52.16 kDa. The GH43 family is expressed in many eukaryotes and prokaryotes, and the evolutionary tree shows that H1AD43 is mainly distributed in species of the genus *Bacillus*, and the function of this protein may be more important for bacteria (Figure 3A). The hydrophobicity prediction results showed that the H1AD43 protein was globally hydrophilic (Figure 3B). The molecular modeling of H1AD43 by SWISS (https://swissmodel.expasy.org/, accessed on 22 July 2021) shows that H1AD43 contains secondary structural units, including β sheets, α helices, β turns, etc. The tertiary structure shows that there is a cavity on the surface of H1AD43, which may be the active site of glycoside hydrolase. As predicted by UniProt (https://www.uniprot.org/, accessed on 27 July 2021) and homologous sequence alignment (Figure S4), the three enzyme active sites of H1AD43 are located at 42Asp, 175Asp, and 228Glu, and all three amino acids are located at holes in the spatial conformation (Figure 3C). The xylanase activity of H1AD43 will be affected to varying degrees by single, double, or triple mutations (Figure S3). It is worth noting that the loss of enzyme activity does not affect the ability of H1AD43 to cause an HR in *N. benthamiana* cells (Figure S3).

### 2.2. H1AD43 Localizes to the Cell Membrane and the Nucleus

**Figure 4:**
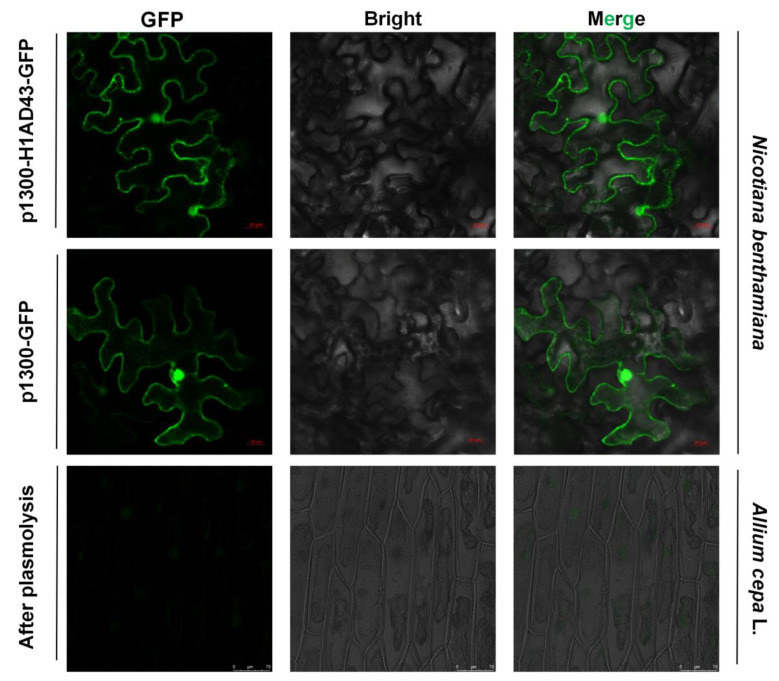
Localization analysis of H1AD43 in *N. benthamiana* and onion epidermal cells.

Plants use cell-surface-located pattern recognition receptors (PRRs) and intracellular leucine-rich receptor nucleotide-binding domains and leucine-rich repeats (NLRs) to sense foreign microorganisms, resulting in the activation of downstream immune signal transmission [27]. Identifying the localization of H1AD43 in cells will help to clarify how this class of glycoside hydrolases functions in cells. The localization of the H1AD43 protein was investigated by the *Agrobacterium*-mediated transient transformation technique. The fluorescence signal was observed by confocal microscopy 48 h after *Agrobacterium* injection. As shown in Figure 4 H1AD43 was localized to the cytoplasm, cell membrane, and nucleus, and it was further demonstrated by plasmolysis of onion epidermal cells that H1AD43 showed no cell wall localization (Figure 4).

### 2.3. H1AD43 Induces a Burst of ROS

**Figure 5:**
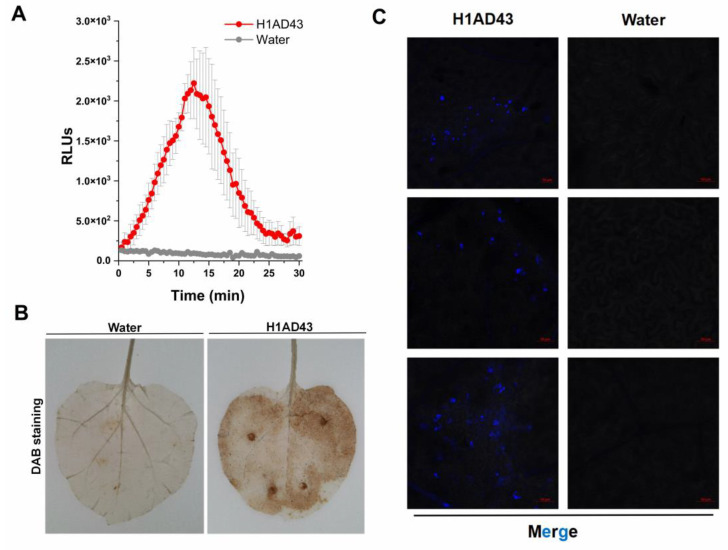
Reactive oxygen species burst and callose deposition. (**A**) H1AD43 triggered an ROS burst in *N. benthamiana* leaves. (**B**) H1AD43 induced the accumulation of ROS in *N. benthamiana* leaves. (**C**) H1AD43 induced callose deposition.

Some cell-death-inducing proteins are often recognized by the plant immune system and act as PAMPs to activate the host PTI response, causing a series of typical features [18]. The ROS burst is one of the early immune responses of plants to resist the invasion of pathogenic bacteria. It can create a superoxic environment in the plant cytoplasm and directly inhibit the growth of pathogenic bacteria [28]. ROS can also act as signaling molecules that induce immune responses in plants [29]. The burst of reactive oxygen species after H1AD43 treatment was analyzed, and the brown precipitate that appeared on *N. benthamiana* leaves after DAB staining proved that abundant O^2−^ was produced in *N. benthamiana* cells (Figure 5A,B). The ROS burst results showed that after H1AD43 was used to treat *N. benthamiana* leaves, the fluorescence intensity reached the highest peak between 10 and 15 min and then began to decrease (Figure 5A), which was also consistent with the trend of ROS burst results in flg22-treated *N. benthamiana* leaves [30].

### 2.4. H1AD43 Promotes Callose Accumulation in N. benthamiana Leaves

Callose is mainly composed of β-D-1,3-glucan and contains phenolic substances [31], which are related to the resistance of plants to various diseases. It can form a thickened cell wall during infection to slow the invasion of pathogens [32]. The accumulation of callose in *N. benthamiana* cells after H1AD43 treatment was quantified and observed by laser confocal microscopy, as shown in Figure 5C. Compared with water treatment, H1AD43 significantly induced the accumulation of callose.

### 2.5. H1AD43 Induces the Upregulation of Defense Genes

**Figure 6:**
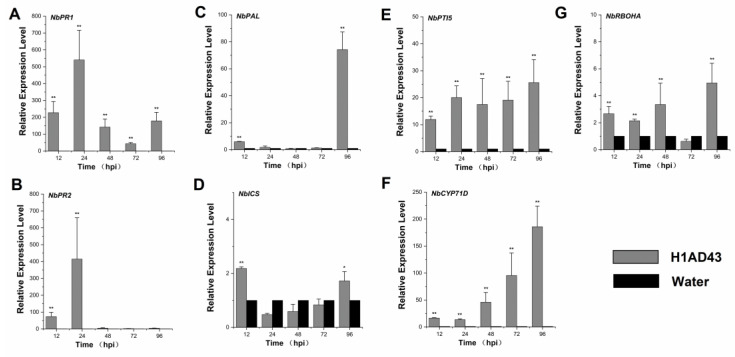
Expression levels of genes related to defense. (**A**) Induction of the disease-course-related gene *NbPR1* in *N. benthamiana* after H1AD43 treatment; (**B**) iInduction of the disease-course-related gene *NbPR*2 in *N. benthamiana* after H1AD43 treatment; (**C**) iInduction of the SA-synthesis-related gene *NbPAL* in *N. benthamiana* after H1AD43 treatment; (**D**) iInduction of the SA-synthesis-related gene *NbICS* in *N. benthamiana* treated with H1AD43; (**E**) iInduction of the PTI gene *NbPTI5* in *N. benthamiana* treated with H1AD43; (**F**) iInduction of the PTI gene *NbCYP71D* in *N. benthamiana* treated with H1AD43; (**G**) iInduction of the ROS-related gene *NbRBOHA* in *N. benthamiana* treated with H1AD43. * represents for *p* < 0.05 and ** represents for *p* < 0.01.

The activation of defense signals in plants mainly manifests in changes in signaling pathways such as the salicylic acid (SA), jasmonic acid (JA), and ethylene (ET) pathways as well as the synthesis of PR proteins [33]. After treatment of *N. benthamiana* leaves with H1AD43, the expression of plant-related defense genes was examined. *NbPR1* and *NbPR2* are related to the synthesis of PR proteins and have been shown to be involved in the systemic defense response of plants, and the expression levels of these two genes peaked at 24 h after treatment of the *N. benthamiana* leaves, which is, respectively, 540- and 414-fold of the controls (Figure 6A,B). The occurrence of induced plant defense responses is often associated with PTI responses, and to prove that H1AD43 is indeed a new protein elicitor, the expression of the PTI marker genes *PTI5* and *CYP71D* was examined. These were also continuously upregulated as treatment time increased: PTI5 was still 8-fold upregulated at 96h after H1AD43 treatment, whereas the upregulated level of CYP71D reached 185-fold (Figure 6E,F). Isochorismate synthase (*ICS*) and phenylalanine ammonia lyase (*PAL*) are two important genes in the plant SA synthesis pathway that are involved in various plant physiological activities and are closely related to plant defense reactions [34]. The *NbPAL* gene was detected to peak at 96 h of H1AD43 treatment, which was 74-fold upregulated relative to the control group (Figure 6C,D). In addition, we quantified the expression level of the ROS-related gene *NbRBOHA*, which was significantly higher than that of the control gene (Figure 6G) at both 48 h and 96 h, which was also consistent with the experimental results regarding the ROS burst.

### 2.6. H1AD43 Induces the Resistance of Tobacco to Phytophthora Capsici and TMV

**Figure 7:**
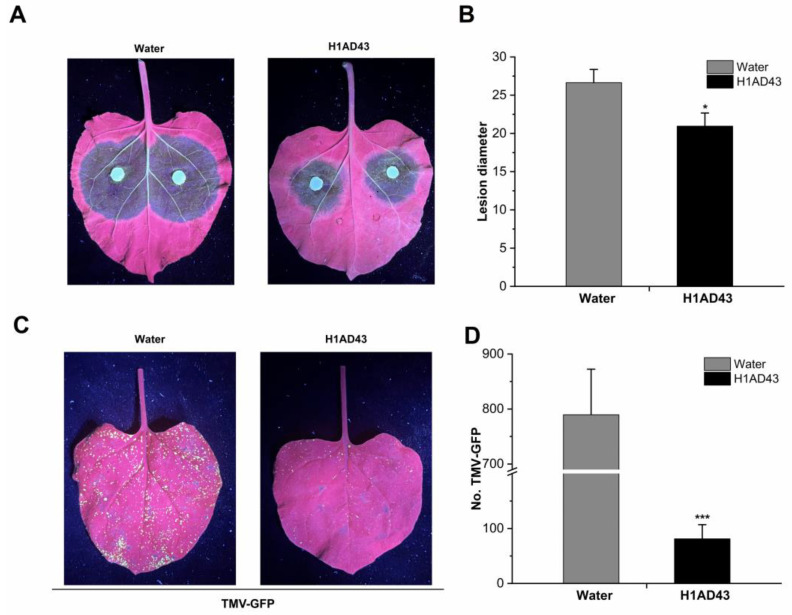
H1AD43 induces resistance to *Phytophthora capsici* and TMV in *N. benthamiana*. (**A**) Symptoms of *Phytophthora* infection; (**B**) Diameter of diseased spots; (**C**) Typical symptoms caused by TMV-GFP in tobacco leaves; (**D**) Number of TMV-GFP diseased spots. * represents for *p* < 0.05 and *** represents for *p* < 0.001.

Elicitors can induce systemic plant resistance to disease, such as FoEG1, an elicitor for promoting cotton resistance to *F. oxysporum* [9]. PeBL1 increases the resistance of *N. benthamiana* to tobacco mosaic virus-green fluorescent protein (TMV-GFP) and *Pseudomonas capsica* [25]. To further confirm the role of H1AD43 in inducing disease resistance in plants, H1AD43 protein was infiltrated into *N. benthamiana* leaves, as shown in Figure 7A,B. After inoculation with *P. capsici*, the disease diameter of the H1AD43-treated group was 20.96 ± 1.7 mm, which was significantly smaller than the control group [35]. The TMV-GFP experiments showed that H1AD43 could effectively inhibit the replication and movement of TMV, and the number of GFP lesions in the H1AD43-treated group was significantly decreased relative to that in the control group (Figure 7C,D).

## 3. Discussion

In the interaction between plants and microorganisms, plants perceive different types of elicitors that induce defense responses, forming the first layer of defense against pathogens [36]. In this study, a new elicitor, H1AD43, from *B. licheniformis* was isolated, and the protein was identified as a member of the glycoside hydrolase 43 family. Glycoside hydrolases play an important role in the infection, pathogenicity, and virulence of pathogens, with the vast majority of reported glycoside hydrolase elicitors derived from pathogenic microorganisms, and a few cases have been reported from PGPR-derived proteins [9,18]. H1AD43 can elicit an HR in *N. benthamiana* leaves; normally, HR-associated cell death is thought to prevent pathogen expansion by cutting off the nutrient source of the pathogen [37]. HR is often associated with PTI responses, and PTI is an early defense response generated by plants after sensing external stimuli [38], so we examined events associated with early defense responses in plants.

The burst of ROS is the most obvious early defense response in plants that can act as signaling molecules and play an important role in regulating plant responses to biotic or abiotic stresses [29]. During the ROS burst, the activities of plasma-membrane-bound NADPH oxidase and cell-wall-bound peroxidase in the plant cytoplasm are enhanced to generate ROS, thereby creating an unsuitable environment for pathogenic bacteria to live in and resist pathogenic bacterial invasion [39]. In this study, a significant ROS burst was observed, along with a significant upregulation of the ROS-related gene *NbRBOHA*.

Elicitor treatment also causes the accumulation of callose in the plant cell wall, resulting in the thickening of the cell wall and forming a physical barrier to the invasion of pathogens [40]. In this study, H1AD43 treatment of *N. benthamiana* induced substantial deposition of callose, suggesting that the protein elicits a plant defense response. The occurrence of PTI responses is usually accompanied by changes in defense signaling pathways in plants, and the PTI marker genes *PTI5* and *CYP71D* are continuously upregulated within 96 h after H1AD43 treatment of *N. benthamiana*. The defense-related genes *NbPR1* and *NbPR2* peaked at 24 h, and ROS production was mediated by the *NbRBOHA* respiratory burst oxidase [41], which was also significantly upregulated after H1AD43 treatment, consistent with the results of reactive oxygen species testing. SA is an important plant hormone involved in various physiological activities, especially related to disease resistance. Isochorismate synthase (*ICS*) and phenylalanine ammonia lyase (*PAL*) are two important genes in the SA synthesis pathway in plants [34], and the *PAL* gene was most upregulated at 96 h after H1AD43 treatment. PGPR itself can induce systemic disease resistance in plants [42]. Most studies have shown that after treating plants with *Bacillus*, induced systemic resistance (ISR) is significantly activated, which can enable plants to produce a broad spectrum of disease resistance to plant pathogens, including fungi, bacteria, viruses, etc. [43]. In this article, we treated *N. benthamiana* with H1AD43 and inoculated the plants with *P. capsici* and TMV. H1AD43 can effectively slow the infection of *P. capsici* on tobacco leaves and inhibit the replication and movement of TMV. These studies further prove that H1AD43 can activate the defense system in *N. benthamiana*.

## 4. Materials and Methods

### 4.1. Materials

*B. licheniformis* (BL06) was isolated from the rhizosphere of healthy cucumber plants [44]. Superdex75 was provided by GE Healthcare Life Science (Piscataway, NJ, USA), and ChamQ Universal SYBR qPCR Master Mix was provided by Vazyme (Nanjing, China). Other chemical reagents were of analytic or chromatographic purity.

### 4.2. Extraction and Purification

BL06 was activated and inoculated in Luria–Bertani (LB, Oxoid, UK) culture medium under oscillation for 72 h (200 rpm, 28 °C). The culture supernatant was cleared of cells by centrifugation (30 min, 10,000× *g*, 4 °C). The supernatant was collected, and ammonium sulfate was slowly added at 4 °C until the saturation reached 90% [25]. After standing overnight, the supernatant was centrifuged, and redissolved with PBS buffer and dialyzed for 48 h. The dialyzed protein was filtered through a 0.22-µm membrane, and the dialyzed protein product was purified by an Akta automatic protein purification system (Akta Avant 25, Boston, MA, USA). The anion column was a HiTrap Capto DEAE column, and the eluents were pure water and NaCl solution. The eluted components were collected, and the HR of these components on *N. benthamiana* leaves was assessed to evaluate their activity. The active fraction was further purified using Superdex 75 (Piscataway, NJ, USA), and the eluted fractions were collected and subjected to activity verification.

### 4.3. H1AD43 Mass Spectrometry Analysis, Gene Cloning, and Expression

A protein sample isolated on a sodium dodecyl sulfate (SDS)-PAGE gel was identified by mass spectrometry (MS) analysis (Beijing Genomics institution, Shenzhen, China). Genomic DNA was isolated from *B. licheniformis* BL06 using a FastPure bacterial DNA kit (Vazyme, Nanjing, China). The primers were designed by analyzing the MS results, and the sequences were cloned from BL06 genomic DNA, cloned into the prokaryotic expression vector pET32a, and then transformed into *Escherichia coli* ArcticExpress (DE3) competent cells (TransGen Biotech, Beijing, China). The recombinant His-tagged protein was obtained by inducing protein expression using 0.1 mM isopropyl β-D-1-thiogaPCRactopyranoside (IPTG, Solarbio, Beijing, China).

The recombinant protein was further purified through a His-Trap HP column (GE Healthcare, Waukesha, WI, USA). Mobile phase A (20 mM Tris-HCl, 500 mM NaCl, 20 mM imidazole, pH 9.0) and mobile phase B (20 mM Tris-HCl, 500 mM NaCl, 250 mM imidazole, pH 9.0) were used to elute the fractions sequentially. The samples were desalted using a 10 kDa ultrafiltration device and were tested for induced *N. benthamiana* leaf HR activity.

### 4.4. H1AD43 Localization

H1AD43 was expressed in *N. benthamiana* leaves using an *Agrobacterium*-mediated transient expression system [45]. In short, the H1AD43 gene was cloned into the transient expression vector pCAMBIA1300 containing a GFP tag. The vector was then transformed into *Agrobacterium* GV3101, and single colonies were picked and cultured overnight in LB liquid medium, after which the *Agrobacterium* cells were resuspended in the infection solution (10 mM MgCl_2_, 10 mM MES, 150 μM acetosyringone, pH = 5.7). The regulatory bacterial suspension (OD_600_ = 1.0) was injected into 6-week-old *N. benthamiana* leaves after standing for 3 h in the dark. The pCAMBIA1300 vector containing only the GFP marker was used as the control group. Confocal observation was performed using a laser confocal microscope (Zeiss LSM 710, Germany) after 48 h. The localization of the H1AD43 protein in the onion epidermis was evaluated according to the experimental method of Xu [46]. The outer 2–3 layers of fresh onions were picked and soaked in 75% alcohol for 5 min and then washed with sterile water. One side of the inner epidermis near the mesophyll was torn off and tiled on MS medium at 25 °C for 24 h. Then, the above *Agrobacterium* was adjusted to OD_600_ = 1.0 with MS liquid medium as the infection solution, and the onion epidermis was immersed in the infection solution for 10 min, tiled in MS solid medium, and cultured at 25 °C for 12–14 h. Cells were treated with 40% sucrose to separate their plasma walls, and laser confocal microscopy was used to visualize the fluorescence.

### 4.5. Measurement of ROS Burst

ROS bursts were detected using both DAB staining and chemiluminescence. After 5-week-old *N. benthamiana* leaves were treated with 1 mg/mL H1AD43 protein, the leaves were stained in DAB dye solution for 8 h according to reported methods [47], decolorized using 90% alcohol, and treated with water as a control. For ROS detection using chemiluminescence [27], five-week-old leaves were selected to make leaf discs using a 4 mm punch and incubated overnight in ddH_2_O. Then, a mixture containing 100 μM luminol, 20 µg/mL horseradish peroxidase, and H1AD43 was added to replace the ddH_2_O, and the fluorescence was immediately measured in a Varioskan LUX multimode microplate reader (Thermo Scientific, Waltham, MA, USA) at 30 s intervals for 30 min.

### 4.6. Detection of Callose

The accumulation of callose was detected according to Mason’s method [48]. After the *N. benthamiana* leaves were treated with 1 mg/mL H1AD43 for 10 h, the leaves were decolorated with an appropriate amount of decolorizing solution (water-saturated phenol:glycerol:water:lactic acid:ethanol = 1:1:1:1:8) to decolorize at 100 ℃, and then the leaves were immersed in aniline blue solution (150 mM K_2_HPO_4_, 0.01% aniline blue) in the dark for 8 h. Confocal observation was performed using a laser confocal microscope (Zeiss LSM 710, Oberkochen, Germany).

### 4.7. Analysis of the H1AD43 Protein Properties

The sequences of the glycoside hydrolase 43 family from fungi, oomycetes, and bacteria were used for homology analysis, and a phylogenetic tree was constructed. The amino acid sequence of H1AD43 and its homologous sequence were obtained from the NCBI database. The hydrophobicity of H1AD43 was predicted using UniProt (https://www.uniprot.org/, accessed on 22 July 2021). The predicted enzyme activity sites were mutated using the Mut Express II Fast Mutagenesis Kit V2 (Vazyme, Nanjing, China). The in vitro enzymatic viability of H1AD43 and mutants thereof was examined according to the method reported by Yi [49].

### 4.8. Gene Expression Analysis of N. benthamiana Treated with H1AD43

To confirm that H1AD43 acts as an elicitor to induce immune defense responses in plants, the expression of the defense genes in *N. benthamiana* leaves was examined after H1AD43 treatment. Total RNA was extracted from *N. benthamiana* leaves at 6 h, 12 h, 24 h, 72 h, and 96 h, and single-stranded cDNA was synthesized using total RNA as the template. The expression levels of defense-related genes were determined by real-time quantitative PCR (RT-qPCR) [50], and *NbEF1α* was used as the reference gene. The specific primer sequences are listed in Table 1.

### 4.9. Detection of H1AD43-Induced Disease Resistance in N. benthamiana Leaves

The induced disease resistance of the H1AD43 protein was tested by expressing the recombinant TMV-GFP virus in *N. benthamiana* leaves using an *Agrobacterium*-mediated transient transformation system [35]. Briefly, 5-week-old *N. benthamiana* leaves were infiltrated with 1 mg/mL H1AD43 and cultured for 24 h at 25 °C in a 16 h light/8 h dark environment. *Agrobacterium* strains carrying the TMV-GFP expression vector were resuspended to OD_600_ = 0.5 and infiltrated into *N. benthamiana* leaves. An ultraviolet lamp was used for subsequent observations after 72 h.

To further identify whether H1AD43 could attenuate pathogen infection, *N. benthamiana* leaves were infiltrated with 1 mg/mL H1AD43 protein, and after 24 h the leaves were inoculated with *P. capsici* and cultured at 25 ℃ for 3 d. Then, the disease spot diameter of each leaf was measured.

## 5. Conclusions

In this study, the glycoside hydrolase H1AD43 was extracted and identified from the fermentation broth of *B. licheniformis*. H1AD43 belongs to the glycoside hydrolase 43 family, which consists of 469 amino acids, and has a molecular weight of approximately 52.16 kD. This protein shows xylan hydrolase activity and can cause an HR in *N. benthamiana*, and the ability to induce an HR does not depend on its enzyme activity. H1AD43 can induce an ROS burst, callose accumulation, defense gene upregulation, and resistance against *P. capsici* and TMV infection in *N. benthamiana*, showing typical elicitor characteristics. This study confirms a glycoside hydrolase class elicitor of *Bacillus*, providing a new theoretical basis and research direction for studying the role of *Bacillus* in promoting disease resistance in plant systems and strongly supporting the potential application of *B. licheniformis* BL06 in biocontrol and sustainable agriculture.

## Figures and Tables

**Table 1:** Primers used for RT-qPCR of defense-related and internal control genes.

Gene Name	Forward Primer	Reverse Primer
*NbPR1*	ATGGTCAATACGGCGAAAAC	CCTAGCACATCCAACACGAA
*NbPR2*	CAACCCGCCCAAAGATAGTA	TCCAAAAGGGCATCAAAAAG
*NbPAL*	GTTATGCTCTTAGAACGTCGCCC	CCGTGTAATGCCTTGTTTCTTGA
*NbICS*	TCATCACTCGTGAAATGGTCG	GAGGCTGGGAGTTAACCAAGT
*NbPTI5*	CCTCCAAGTTTGAGCTCGGATAGT	CCAAGAAATTCTCCATGCACTCTGTC
*NbCYP71D*	AAGGTCCACCGCACCATGTCCTTAGAG	AAGAATTCCTTGCCCCTTGAGTACTTGC
*NbRBOHA*	CGTGCTTGATAAAGAAACACTGA	CCCACCCAACCAAAATACGC
*NbEF1α*	AGCTTTACCTCCCAAGTCATC	AGAACGCCTGTCAATCTTGG

## Data Availability

The data presented in this study are available on request from the corresponding author.

## Supplementary Materials

The following supporting information can be downloaded at: https://www.mdpi.com/article/10.3390/ijms232214435/s1.

## References

[1] Trdá L., Boutrot F., Claverie J., Brulé D., Dorey S., Poinssot B. (2015). Perception of pathogenic or beneficial bacteria and their evasion of host immunity: Pattern recognition receptors in the frontline. Front Plant Sci..

[2] Saijo Y., Loo E.P., Yasuda S. (2018). Pattern recognition receptors and signaling in plant-microbe interactions. Plant J..

[3] Yuan M., Ngou B.P.M., Ding P., Xin X.F. (2021). PTI-ETI crosstalk: An integrative view of plant immunity. Curr. Opin. Plant Biol..

[4] Barghahn S., Arnal G., Jain N., Petutschnig E., Brumer H., Lipka V. (2021). Mixed Linkage β-1,3/1,4-Glucan Oligosaccharides Induce Defense Responses in *Hordeum vulgare* and *Arabidopsis thaliana*. Front Plant Sci..

[5] Zhang J., Zhou J.-M. (2010). Plant immunity triggered by microbial molecular signatures. Mol. Plant..

[6] Chinchilla D., Bauer Z., Regenass M., Boller T., Felix G. (2006). The *Arabidopsis* receptor kinase FLS2 binds flg22 and determines the specificity of flagellin perception. Plant Cell..

[7] Kunze G., Zipfel C., Robatzek S., Niehaus K., Boller T., Felix G. (2004). The N terminus of bacterial elongation factor Tu elicits innate immunity in *Arabidopsis* plants. Plant Cell..

[8] Nürnberger T., Nennstiel D., Jabs T., Sacks W.R., Hahlbrock K., Scheel D. (1994). High affinity binding of a fungal oligopeptide elicitor to parsley plasma membranes triggers multiple defense responses. Cell.

[9] Zhang L., Yan J.-P., Fu Z.-C., Shi W.-J., Ninkuu V., Li G.-Y., Yang X.-F., Zeng H.-M. (2021). FoEG1, a secreted glycoside hydrolase family 12 protein from *Fusarium oxysporum*, triggers cell death and modulates plant immunity. Mol. Plant Pathol..

[10] Felix G., Boller T. (2003). Molecular sensing of bacteria in plants. The highly conserved RNA-binding motif RNP-1 of bacterial cold shock proteins is recognized as an elicitor signal in tobacco. J. Biol. Chem..

[11] Erbs G., Newman M.A. (2012). The role of lipopolysaccharide and peptidoglycan, two glycosylated bacterial microbe-associated molecular patterns (MAMPs), in plant innate immunity. Mol. Plant Pathol..

[12] Ramonell K., Berrocal-Lobo M., Koh S., Wan J., Edwards H., Stacey G., Somerville S. (2005). Loss-of-function mutations in chitin responsive genes show increased susceptibility to the powdery mildew pathogen *Erysiphe cichoracearum*. Plant Physiol..

[13] Naumoff D.G. (2011). Hierarchical classification of glycoside hydrolases. Biochemistry.

[14] Miedes E., Vanholme R., Boerjan W., Molina A. (2014). The role of the secondary cell wall in plant resistance to pathogens. Front. Plant Sci..

[15] Cosgrove D.J. (2005). Growth of the plant cell wall. Nat. Rev. Mol. Cell Biol..

[16] Yang Y., Yang X., Dong Y., Qiu D. (2018). The *Botrytis cinerea* Xylanase BcXyl1 Modulates Plant Immunity. Front Microbiol..

[17] Kubicek C.P., Starr T.L., Glass N.L. (2014). Plant cell wall-degrading enzymes and their secretion in plant-pathogenic fungi. Annu. Rev. Phytopathol..

[18] Ma Z.-C., Song T.-Q., Zhu L., Ye W.-W., Wang Y., Shao Y.-Y., Dong S.-M., Zhang Z.-G., Dou D.-L., Zheng X.-B. (2015). A *Phytophthora sojae* Glycoside Hydrolase 12 Protein Is a Major Virulence Factor during Soybean Infection and Is Recognized as a PAMP. Plant Cell.

[19] Yin Z.-Y., Wang N., Pi L., Li L., Duan W.-W., Wang X.-D., Dou D.-L. (2021). *Nicotiana benthamiana* LRR-RLP NbEIX2 mediates the perception of an EIX-like protein from *Verticillium dahliae*. J. Integr. Plant Biol..

[20] Frías M., González M., González C., Brito N. (2019). A 25-Residue Peptide From *Botrytis cinerea* Xylanase BcXyn11A Elicits Plant Defenses. Front Plant Sci..

[21] Furman-Matarasso N., Cohen E., Du Q., Chejanovsky N., Hanania U., Avni A. (1999). A point mutation in the ethylene-inducing xylanase elicitor inhibits the beta-1-4-endoxylanase activity but not the elicitation activity. Plant Physiol..

[22] Akhtar S.S., Amby D.B., Hegelund J.N., Fimognari L., Großkinsky D.K., Westergaard J.C., Müller R., Moelbak L., Liu F., Roitsch T. (2020). *Bacillus licheniformis* FMCH001 Increases Water Use Efficiency via Growth Stimulation in Both Normal and Drought Conditions. Front Plant Sci..

[23] Ongena M., Jourdan E., Adam A., Paquot M., Brans A., Joris B., Arpigny J.L., Thonart P. (2007). Surfactin and fengycin lipopeptides of *Bacillus subtilis* as elicitors of induced systemic resistance in plants. Environ. Microbiol..

[24] Wang N.-B., Liu M.-J., Guo L.-H., Yang X.-F., Qiu D.-W. (2016). A Novel Protein Elicitor (PeBA1) from *Bacillus amyloliquefaciens* NC6 Induces Systemic Resistance in Tobacco. Int. J. Biol. Sci..

[25] Wang H.-Q., Ynag X.-F., Guo L.-H., Zeng H.-M., Qiu D.-W. (2015). PeBL1, a novel protein elicitor from *Brevibacillus laterosporus* strain A60, activates defense responses and systemic resistance in *Nicotiana benthamiana*. Appl. Environ. Microbiol..

[26] Pitsili E., Phukan U.J., Coll N.S. (2020). Cell Death in Plant Immunity. Cold Spring Harb. Perspect. Biol..

[27] Nie J.-J., Yin Z.-Y., Li Z.-P., Wu Y.-X., Huang L.-L. (2019). A small cysteine-rich protein from two kingdoms of microbes is recognized as a novel pathogen-associated molecular pattern. New Phytol..

[28] Waszczak C., Carmody M., Kangasjärvi J. (2018). Reactive Oxygen Species in Plant Signaling. Annu. Rev. Plant Biol..

[29] Mhamdi A., Van Breusegem F. (2018). Reactive oxygen species in plant development. Development.

[30] Yang Q., Huai B., Lu Y., Cai K., Guo J., Zhu X., Kang Z., Guo J. (2020). A stripe rust effector Pst18363 targets and stabilises TaNUDX23 that promotes stripe rust disease. New Phytol..

[31] Apostolakos P., Giannoutsou E., Galatis B. (2021). Callose: A multifunctional (1, 3)-β-DD-glucan involved in morphogenesis and function of angiosperm stomata. J. Biol. Res..

[32] Naumann M., Somerville S., Voigt C. (2013). Differences in early callose deposition during adapted and non-adapted powdery mildew infection of resistant *Arabidopsis* lines. Plant Signal Behav..

[33] Di X., Gomila J., Takken F.L.W. (2017). Involvement of salicylic acid, ethylene and jasmonic acid signalling pathways in the susceptibility of tomato to *Fusarium oxysporum*. Mol Plant Pathol..

[34] An C., Mou Z. (2011). Salicylic acid and its function in plant immunity. J. Integr. Plant Biol..

[35] Liu C.-Y., Tian S.-R., Lv X., Pu Y.-D., Peng H.-R., Fan G.-J., Ma X.-Z., Ma L.-S., Sun X.-C. (2022). *Nicotiana benthamiana* asparagine synthetase associates with IP-L and confers resistance against tobacco mosaic virus via the asparagine-induced salicylic acid signalling pathway. Mol. Plant Pathol..

[36] Zipfel C. (2009). Early molecular events in PAMP-triggered immunity. Curr. Opin. Plant Biol..

[37] Balint-Kurti P. (2019). The plant hypersensitive response: Concepts, control and consequences. Mol Plant Pathol..

[38] Chisholm S.T., Coaker G., Day B., Staskawicz B.J. (2006). Host-microbe interactions: Shaping the evolution of the plant immune response. Cell.

[39] Kadota Y., Shirasu K., Zipfel C. (2015). Regulation of the NADPH Oxidase RBOHD During Plant Immunity. Plant Cell Physiol..

[40] Zavaliev R., Ueki S., Epel B.L., Citovsky V. (2011). Biology of callose (β-1,3-glucan) turnover at plasmodesmata. Protoplasma.

[41] Zhang H.-J., Fang Q., Zhang Z.-G., Wang Y.-C., Zheng X.-B. (2009). The role of respiratory burst oxidase homologues in elicitor-induced stomatal closure and hypersensitive response in *Nicotiana benthamiana*. J. Exp. Bot..

[42] Zhu L., Huang J.-M., Lu X.-M., Zhou C. (2022). Development of plant systemic resistance by beneficial rhizobacteria: Recognition, initiation, elicitation and regulation. Front Plant Sci..

[43] Wang H., Liu R.-J., You M.P., Barbetti M.J., Chen Y. (2021). Pathogen Biocontrol Using Plant Growth-Promoting Bacteria (PGPR): Role of Bacterial Diversity. Microorganisms.

[44] Liu X.-Y., Bao T.-T., Zheng L., Kgosi V.T., Liu H.-X. (2021). Cell wall integrity in *Magnaporthe oryzae* is weakened by proteins secreted by *Bacillus licheniformis* BL06. Biol. Control.

[45] Cui Y., Gao C.-J., Zhao Q., Jiang L.-W. (2016). Using Fluorescent Protein Fusions to Study Protein Subcellular Localization and Dynamics in Plant Cells. Methods Mol. Biol..

[46] Xu K.-D., Huang X.-H., Wu M.-M., Wang Y., Chang Y.-X., Liu K., Zhang J., Zhang Y., Zhang F.-L., Yi L.-M. (2014). A rapid, highly efficient and economical method of Agrobacterium-mediated in planta transient transformation in living onion epidermis. PLoS ONE.

[47] Thordal-Christensen H., Zhang Z.-G., Wei Y.-D., Collinge D.B. (1997). Subcellular localization of H_2_O_2_ in plants. H_2_O_2_ accumulation in papillae and hypersensitive response during the barley-powdery mildew interaction. Plant J..

[48] Mason K.N., Ekanayake G., Heese A. (2020). Staining and automated image quantification of callose in *Arabidopsis* cotyledons and leaves. Methods Cell Biol..

[49] Yi Z.-W., Cai Z.-W., Zeng B., Zeng R.-Y., Zhang G.-Y. (2020). Identification and Characterization of a Novel Thermostable and Salt-Tolerant β-1,3 Xylanase from *Flammeovirga pacifica* Strain WPAGA1. Biomolecules.

[50] Franco-Orozco B., Berepiki A., Ruiz O., Gamble L., Griffe L.L., Wang S., Birch P., Kanyuka K., Avrova A. (2017). A new proteinaceous pathogen-associated molecular pattern (PAMP) identified in *Ascomycete fungi* induces cell death in Solanaceae. New Phytol..

