# Supplementary material for: A Bacillus licheniformis Glycoside Hydrolase 43 Protein Is Recognized as a MAMP
Source: Int J Mol Sci. 2022 Nov 20;23(22):14435. doi: 10.3390/ijms232214435 (PMC9697650; doi:10.3390/ijms232214435)
Supplement: Supplementary file 1 [file ijms-23-14435-s001.zip › ijms-2009869-supplementary.pdf]

Supplementary Figures

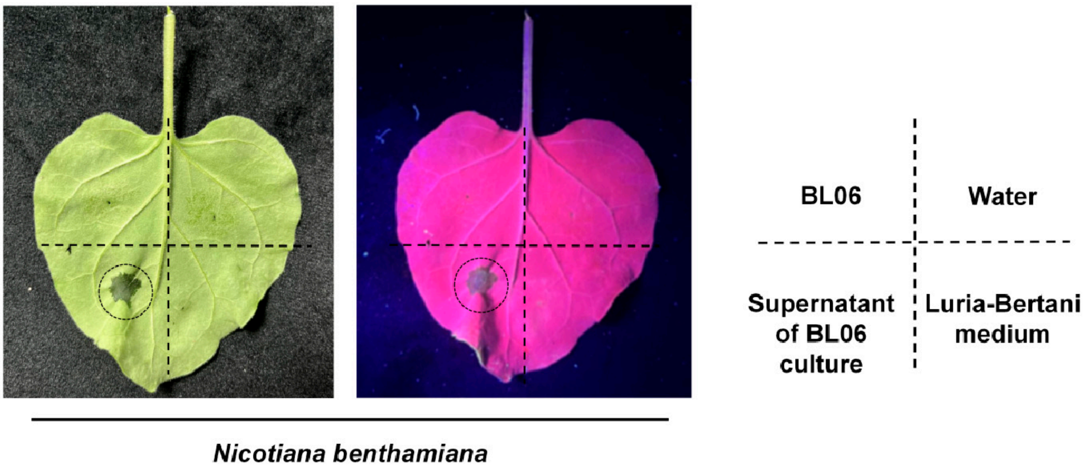

Figure S1. Supernatant of *Bacillus licheniformis* (BL06) fermentation broth can induce HR response in *Nicotiana benthamiana* leaves.

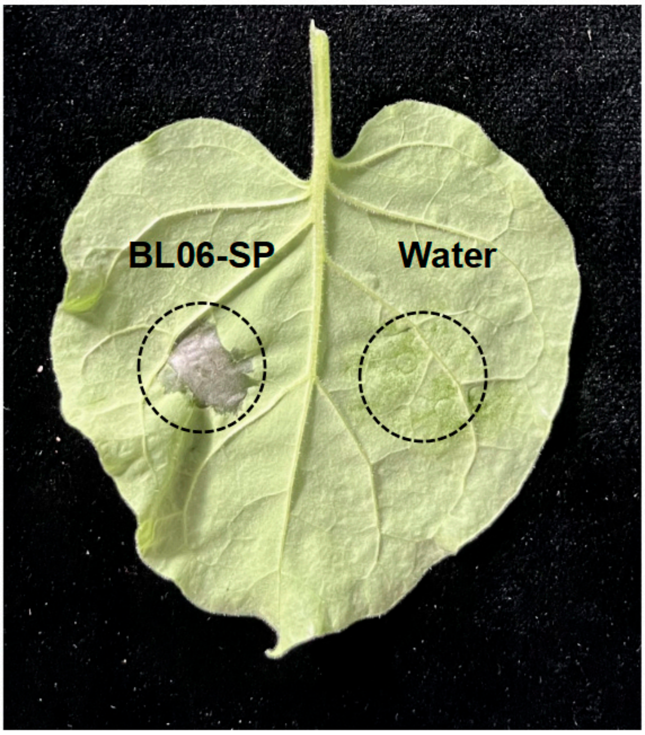

Figure S2. Crude protein of *B. licheniformis* (BL06) can induce HR response in *N. benthamiana* leaves.

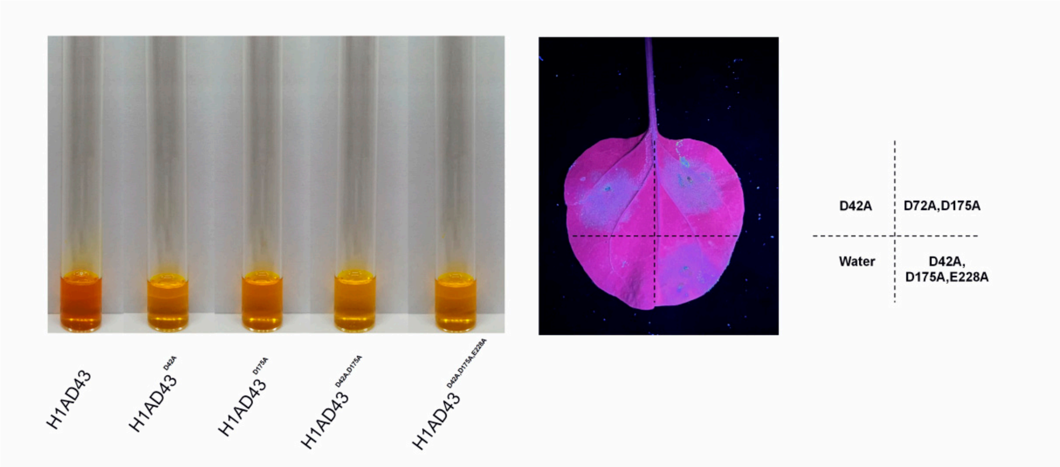

Figure S3. Determination of recombinant H1AD43 enzyme activity and its active site mutation.

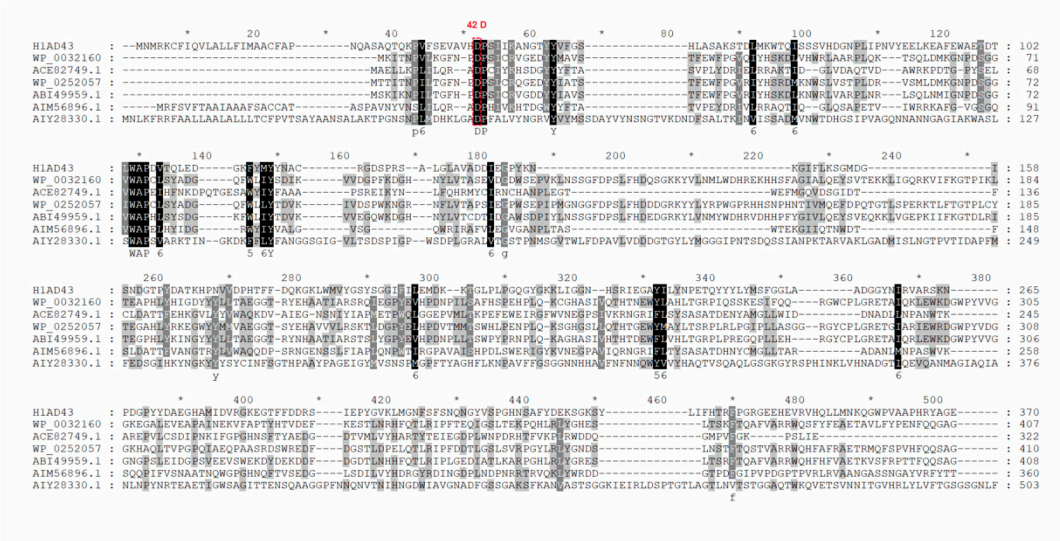

Figure S4. Amino acid sequence alignment of H1AD43 with members of the glycoside hydrolyase 43 family.
